# Supportive enriched environment improves recovery from persistent motor and cognitive impairments after severe traumatic brain injury

**DOI:** 10.3389/fnbeh.2025.1696641

**Published:** 2026-01-12

**Authors:** Margaret Anne Lovier, Michele Kyle, Karen Hughes, Li-Ru Zhao

**Affiliations:** Department of Neurosurgery, The State University of New York Upstate Medical University, Syracuse, NY, United States

**Keywords:** chronic phase, enriched environment, neurological function recovery, severe TBI, supportive enriched environment

## Abstract

**Objective:**

Severe traumatic brain injury (sTBI) causes permanent disability in adults worldwide. While enriched environments (EE) have been shown to improve recovery in the early post-TBI period, their efficacy during the chronic phase of sTBI remains unclear. This study evaluated neurological function recovery in mice with chronic sTBI housed in either traditional EE or supportive EE.

**Methods:**

Adult male C57BL mice were subjected to sTBI by controlled cortical impact and maintained in standard environments (SE) for 7 months. sTBI mice were then randomized into SE (TBI-SE), traditional EE (TBI-EE-1), or supportive EE (TBI-EE-2, co-housed with sham mice). Sham controls were housed in SE (Sham-SE) or supportive EE (Sham-EE-2). EE consisted of a large stainless-steel cage with toys replaced three times weekly. Mice remained in these conditions for 10 weeks, and neurobehavioral testing was performed beginning in week 6.

**Results:**

In the RotaRod test, TBI-SE mice displayed persistent motor coordination and learning deficits, whereas TBI-EE-2 mice showed robust motor coordination recovery and improved motor learning. Of all TBI mice, only the TBI-EE-2 mice demonstrated improved motor learning. In the Morris water maze test, both TBI-EE-1 and TBI-EE-2 groups showed enhanced spatial learning and memory compared with TBI-SE. Y-maze testing revealed impaired short-term memory in TBI-EE-1 mice but significant improvement in TBI-EE-2 mice. Anxiety-like behavior, assessed by open field and light–dark box tests, was reduced only in the TBI-EE-2 mice.

**Conclusion:**

Supportive EE more effectively reduced anxiety and improved motor and cognitive function in chronic sTBI compared with conventional EE. These findings highlight the potential value of incorporating social integration with healthy individuals into rehabilitation programs to optimize recovery in chronic severe TBI.

## 1 Introduction

Traumatic brain injury (TBI) is a major global health concern and a leading causes of long-term disability and death worldwide (Dams-O’Connor et al., 2023; Robinson, 2021). Each year, an estimated 50 million people sustain a TBI, indicating that up to half of the world’s population will experience one TBI during their lifetimes (Dams-O’Connor et al., 2023; Khellaf et al., 2019). The resulting mortality, disability, and healthcare costs impose significant economic burdens worldwide (Dams-O’Connor et al., 2023; Khellaf et al., 2019), and past estimates of the total lifetime cost in the United States have been as high as $221 billion while likely still underestimating the downstream effects (Coronado et al., 2012). Severe TBIs (sTBIs) account for a substantial portion of this burden, as they are the leading cause of death and lifelong disability in children and an increasingly common outcome among older adults due to ground-level falls (Sundstrøm et al., 2020). Case fatality following sTBI approaches 50% and may reach 68% in older women (Robinson, 2021; Sundstrøm et al., 2020). Although sTBI is typically diagnosed using the Glasgow Coma Scale, outcomes are highly heterogeneous in both acute and chronic phases. This variability reflects the diverse causes of trauma, multiple mechanisms of primary and secondary neuronal injury, disparities in access to neurosurgical and intensive care, and individual differences in baseline brain health and plasticity (Dams-O’Connor et al., 2023; Robinson, 2021; Khellaf et al., 2019; Sundstrøm et al., 2020). Overall, sTBI represents one of the most complex disorders of the body’s most complex organ (Sundstrøm et al., 2020).

Although the current standards of care manage TBI primarily as an acute condition, a paradigm shift over the past decade has recognized TBI as a chronic condition, with lifelong consequences affecting approximately 75% of patients (Dams-O’Connor et al., 2023; Robinson, 2021; Khellaf et al., 2019; Sundstrøm et al., 2020; Maegele et al., 2015). Individuals with TBI, particularly those with sTBI, face increased risk for long-term functional disability, cognitive impairment, psychiatric disorders (including substance misuse), neuroendocrine dysfunction, metabolic dysregulation, and cardiovascular disease, among sequelae; these complications may continue to evolve for years after the initial injury (Dams-O’Connor et al., 2023; Sundstrøm et al., 2020). Despite this, therapeutic options for the chronic phase of TBI remain limited (Robinson, 2021; Khellaf et al., 2019; Sundstrøm et al., 2020). Most current treatments focus on reducing secondary injury in the acute phase and managing complications during neurosurgical and intensive care, while public health efforts emphasize primary prevention (Robinson, 2021; Khellaf et al., 2019; Sundstrøm et al., 2020). Pharmacological interventions for TBIs have been investigated for decades, and although several candidate agents show promise, none have yet been approved or broadly recommended for clinical use (Khellaf et al., 2019; Sundstrøm et al., 2020). As a result, intensive rehabilitation and long-term clinical follow-up remain the cornerstone of chronic TBI care, even in cases of sTBIs (Sundstrøm et al., 2020; Schreiber et al., 2014). Notably, high-quality, continuous rehabilitation initiated early after TBI has been shown to improve functional outcomes, enhance quality of life, reduce healthcare costs, and support community reintegration (Sundstrøm et al., 2020).

Rehabilitation has also been shown to be one of the most consistently effective interventions for TBI in preclinical studies. In rodent models, rehabilitation is often simulated through enriched environments (EEs). Unlike standard housing, EEs are larger, accommodate more animals, and include diverse objects of varying size, shape, color, and texture to promote mental, social, and physical stimulation (Bondi et al., 2014). EEs were first introduced in the 1940s as a tool to enhance problem-solving in healthy rats; EEs have since been shown to promote plasticity and improve neurobehavioral outcomes across numerous neurological conditions, including Alzheimer’s diseases, Parkinson’s disease, spinal cord injury, stroke, and TBI (Bondi et al., 2014). Current evidence strongly supports EE both as a preclinical model of neurorehabilitation and as an effective intervention in its own right for TBI recovery, with demonstrated benefits at behavioral, neurochemical, and structural levels (Maegele et al., 2015; Schreiber et al., 2014; Bondi et al., 2014; Dijkhuizen et al., 2024; Kovesdi et al., 2011). In particular, EE has been shown to enhance neural plasticity and induce molecular adaptations in the hippocampus, prefrontal cortex, amygdala, and locus coeruleus (Dijkhuizen et al., 2024).

However, the majority of previous studies have treated TBI as an acute condition, introducing EE either immediately or within days after the injury. Few studies have extended symptom tracking beyond the first 2–4 weeks post-TBI, despite evidence that rodents may take 2 months or longer to reach maximum recovery (Maegele et al., 2015; Kovesdi et al., 2011). Even those that evaluate later outcomes often begin EE exposure immediately after TBI, modeling an ideal clinical scenario of timely neurorehabilitative intervention. In reality, many patients may not receive optimal acute care, may continue to experience evolving or worsening symptoms over time, or may still seek additional recovery opportunities well after discharge from initial treatment (Sundstrøm et al., 2020; Maegele et al., 2015; Bondi et al., 2014; Kovesdi et al., 2011). Compounding this issue, most EE studies have focused on mild TBI rather than severe TBI, limiting their relevance for the patient populations most affected (Schreiber et al., 2014; Bondi et al., 2014).

Although EE has been widely investigated as a rehabilitative strategy after TBI, nearly all published studies apply EE during the early phase, beginning immediately or within the first 7 days after injury. In rodent models of controlled cortical impact or blast TBI, early EE has been shown to improve motor and cognitive recovery, reduce neuroinflammation, and increase hippocampal neuron survival (Kovesdi et al., 2011; Hoffman et al., 2008; Tapias et al., 2022; Bittner et al., 2025). Despite these reported benefits, prior work has focused almost exclusively on early post-injury windows, leaving the chronic phase (>6 months) largely unexplored, particularly in the context of severe TBI. Consequently, it remains unknown whether long-term, progressive neurological deficits are reversible when intervention is delayed to clinically relevant, chronic time points. Our study addresses this critical gap by initiating EE at 7 months post-TBI in a severe controlled cortical impact model to determine whether chronic neurological deficits remain modifiable long after injury.

Because most patients with severe TBI experience delayed access to rehabilitation and continue living in standard environments for many months or even years, we housed mice in standard conditions for 7 months before introducing EE to better model this chronic clinical reality. Long-term studies have shown that standard housing after controlled cortical impact-induced severe TBI fails to reverse persistent and progressive brain tissue loss, white matter degeneration, and permanent deficits in somatosensory, motor, and cognitive function, which have been documented from the acute phase through the chronic phase, lasting from days to weeks, months, and even up to one year post-injury (Pischiutta et al., 2018; Toshkezi et al., 2018; Qiu et al., 2020; Qiu et al., 2023). These findings indicate that chronic neurological deficits persist without intervention. Therefore, our goal was to determine whether delayed EE can improve neurological recovery when initiated long after injury.

In the present study, EE sTBI mice were divided into two disparate experimental groups: one housed exclusively with other sTBI mice, and another housed with sham mice. The latter group, referred to as *supportive EE*, was designed to promote healthy social interaction and play with uninjured peers. A previous stroke study showed that pairing one sham mouse with one stroke mouse immediately after ischemic injury enhanced neurogenesis and improved motor function recovery (Venna et al., 2014). However, studies that house injured and uninjured animals together within enriched environments remain scarce. Our study is designed to address this understudied area.

The overall objective of this study was to evaluate whether EE and supportive EE can improve neurological recovery in the chronic phase of severe TBI.

## 2 Methods

All methods were carried out in accordance with the National Institutes of Health Guide for the Care and Use of Laboratory Animals and approved by the State University of New York Upstate Medical University Institutional Animal Care and Use Committee.

### 2.1 Controlled cortical impact TBI model

Young adult male C57BL mice (3 months old, Jackson Laboratory, Bar Harbor, ME, United States) were subjected to TBI. The controlled cortical impact (CCI) model for severe TBI in young adult mice was performed as described in our previous studies (Qiu et al., 2020; Qiu et al., 2021). Briefly, mice were anesthetized with Avertin (0.4 g/kg, i.p., Sigma-Aldrich, St. Louis, MO, United States) and immobilized in a stereotaxic frame (Leica Biosystems Inc., Germany). Following a midline scalp incision, the skull was exposed, and a 4-mm-diameter craniotomy was created 2 mm lateral to bregma on the right side of the skull. After exposing the dura, an impactor (Impact One Stereotaxic Impactor; Leica Biosystems, Wetzlar, Germany) with a 3-mm flat tip at a 4° angle was positioned at the center of the craniotomy. Severe TBI was induced using an electromagnetically driven impact at 2-mm impact depth, 1.5 m/s velocity, and 8.5-s dwell time. Sham mice underwent the same procedure except for the craniotomy and impact. The scalp incision was closed with 3-0 Prolene sutures, and mice were placed on a homeothermic heating pad (37 °C) to prevent post-anesthesia hypothermia. All mice received ampicillin (20–100 mg/kg, s.c.) and sustained-release buprenorphine (0.6 mg/kg, s.c.) to minimize postoperative infection and pain.

Our previous studies have validated this CCI paradigm as a severe TBI model, characterized by persistent and progressive neuropathology and long-lasting somatosensory, motor, and cognitive function deficits persisting for weeks, months, and even beyond one-year post-injury (Toshkezi et al., 2018; Qiu et al., 2020; Qiu et al., 2023).

### 2.2 Housing conditions

For the enriched environment (EE) condition, mice were housed in a large, round stainless-steel cage (122 cm in diameter, 61 cm in height) with a mesh lid for proper ventilation and aspen wood-chip bedding. The EE cages contained a variety of plastic and metal enrichment objects of different colors and textures, including, but not limited to, running wheels, tunnels, hideouts, and ladders. Objects were replaced three times per week to maximize novelty and stimulation. For the standard environment (SE) condition, mice were housed in a standard polycarbonate mouse cage (4–5 mice per cage; 19 cm × 29 cm × 13 cm) lined with wood-chip bedding and containing no enrichment objects.

### 2.3 Experimental groups

**Figure 1:**
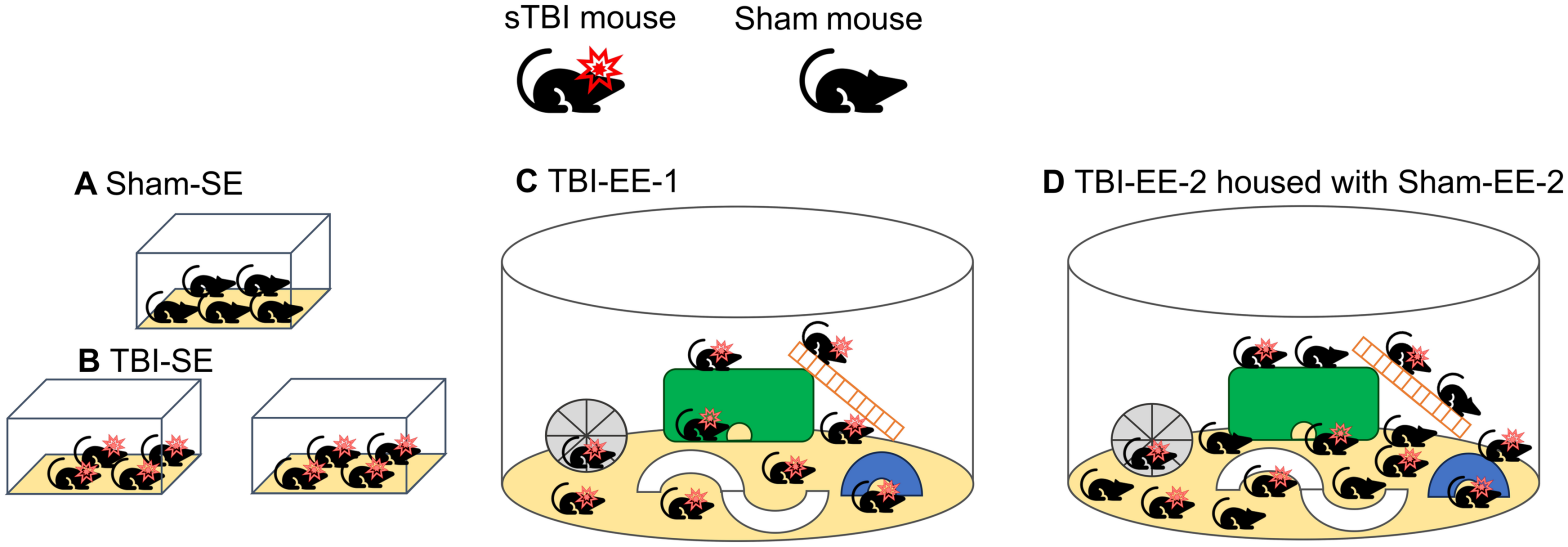
Schematic diagram illustrating housing conditions and group assignments. TBI mice were allocated to standard environments (SE; TBI-SE), enriched environments (EE-1; TBI-EE-1), or supportive enriched environments (EE-2; TBI-EE-2), while sham controls were housed in either SE (Sham-SE) or EE-2 (Sham-EE-2). **(A)** Sham mice housed under SE conditions. **(B)** TBI mice housed under SE conditions. **(C)** TBI mice housed under EE-1 conditions (TBI mice only). **(D)** TBI mice housed under supportive EE-2 conditions (TBI mice co-housed with sham mice).

**Figure 2:**
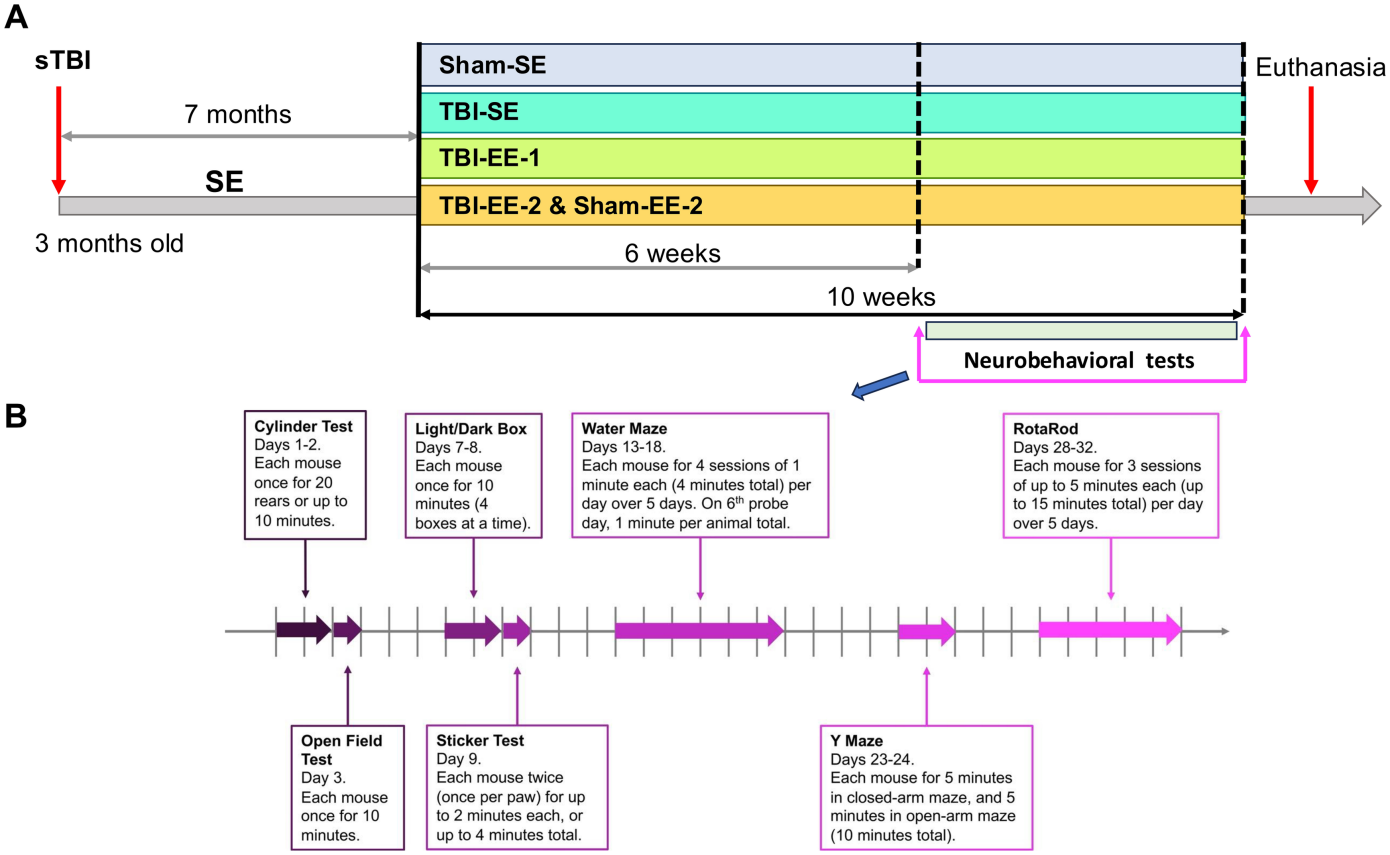
Experimental flowchart illustrating the study design. **(A)** The timeline shows induction of severe traumatic brain injury (sTBI), housing in standard environments (SE) for up to 7 months post-sTBI, initiation of different housing conditions, subsequent group assignments to SE or enriched environments (EE), the duration of the housing period, and when the behavioral assessments were performed. **(B)** The timeline describes in further detail the days and order of each neurobehavioral test.

After recovery from surgery, mice then returned to their standard environments for 7 months before being randomly assigned to remain in the SE or enter the EE (Figures 1, 2). Sham animals were randomized to the standard environment group (Sham-SE, *n* = 5) or the supportive enriched environment group (Sham-EE-2, *n* = 7). TBI animals were randomly assigned to one of the three experimental groups: (1) a standard environment group (TBI-SE, *n* = 9; 4–5 mice per cage); (2) a traditional enriched environment group (TBI-EE-1, *n* = 9), in which TBI mice were the only occupants of the enriched environment cage; and (3) a supportive enriched environment group (TBI-EE-2, *n* = 9), in which TBI mice were housed in the same enriched environment cage with Sham-EE-2 mice.

Four TBI mice in the TBI-EE-1 environment experienced injuries from other TBI mice. These injured TBI mice ultimately necessitated their physical separation from the other TBI-EE-1 mice but remained in the same EE cage. These 4 mice were placed across 2 metal cages (2 per cage) inside the TBI-EE-1 large cage and supplied with a running wheel and at least one additional enrichment object for their continued physical, mental, and social stimulation. In accordance with the same procedure, one mouse was isolated in a metal cage in the TBI-EE-2/Sham-EE-2 large cage due to the presence of an injury wound. One mouse from the TBI-SE group was excluded from the analysis because of a very minor lesion detected after brain collection, resulting in a final sample size of 8 for this group.

All mice were housed under a 12 h light/dark cycle (Light hours: 6:30 a.m. to 6:30 p.m. Dark hours: 6:30 p.m. to 6:30 a.m.) with ad libitum access to water and a standard laboratory diet.

### 2.4 Neurobehavioral tests

After 6 weeks in the randomized standard and enriched environments, the mice underwent neurobehavioral testing over the course of 4 weeks. All testing was performed from 6 a.m. to 1 p.m. During behavioral testing, mice remained in their original environment. (i.e., either standard or enriched environments). At the end of the behavioral testing (i.e., 10 weeks total in the assigned environments), the animals were anesthetized with ketamine (100 mg/kg, i.p.) and xylazine (10 mg/kg, i.p.) and euthanized with transcardiac perfusion. One TBI-SE mouse was ultimately excluded from the final data analysis for not sustaining a sufficiently severe TBI upon postmortem analysis, bringing the total TBI-SE sample down to 8 mice.

The ANY-Maze Video Tracking System (Stoelting Co., Wood Dale, IL, United States) was used for recording mouse performance during the tests.

#### 2.4.1 Cylinder test

The cylinder or paw drag test is a validated measure of functional deficits in forelimb activity among mice (Roome and Vanderluit, 2015). Asymmetry in both exploratory forelimb touches against the cylinder wall and in dragging motions of the forepaws can indicate sensorimotor cortex damage and resulting impairment for contralateral limbs (Roome and Vanderluit, 2015). To perform the test, mice were placed in a clear plexiglass cylinder (9.5 cm in diameter and 44 cm tall) on a clear-topped table after acclimating to the behavioral testing room for 30 min. Their behavior in the cylinder was recorded by a camera below the table for up to 10 min in order to record at least 20 rears, and the captured video was subsequently analyzed to capture the number of forepaw touches and drags on both ipsilateral and contralateral sides to the lesion. Drags were calculated as a percentage of paw touches per side.

#### 2.4.2 Open field maze

The open field maze test is one of the oldest and most widely used neurobehavioral tests for rodents, at least partially due to its simplicity (Seibenhener and Wooten, 2015). The open field test involves an open-topped square box measuring 45 cm × 45 cm × 40 cm, into the center of which the mouse is placed at the start of the test. The mouse is then allowed to roam the open field for 5 min while a video camera above the field tracks its movement. The open field test measures anxiety-related behaviors, such as thigmotaxis and locomotion (Seibenhener and Wooten, 2015).

#### 2.4.3 Light–dark box

Similar to the open field test, the light–dark box test relies on mice’s natural aversion to open, brightly lit spaces (Bourin and Hascoët, 2003). This aversion is believed to increase with anxiety, and thus a mouse with greater baseline anxiety would hypothetically spend a greater proportion of time in a small dark space than it would exploring a larger bright space (Bourin and Hascoët, 2003). The light–dark box test applies this phenomenon by dividing an open field maze into an open and well-lit section, approximately two-thirds of the total area, and a smaller section with opaque walls and ceiling to ensure darkness inside. A small opening in the central wall allows passage between the light and dark areas. The time in each section, light and dark, is recorded for 10 min, as is the number of times the mouse’s head passes through the opening (peeks).

#### 2.4.4 Sticker test

The sticker or adhesive tape removal test is another validated test used to demonstrate somatosensory and motor function among a variety of animals, including mice (Bouet et al., 2009). Because it measures each paw individually (affected and unaffected) and encompasses both a mouse’s ability to sense the sticker and then have the fine motor skills to remove it, the sticker test can be an important multidimensional tool to measure contralateral and ipsilateral cortical damage (Bouet et al., 2009). To conduct the test, the mouse was removed from their home cage and placed in the testing container (1,000 mL plastic beaker) for a habituation period of 60s. A 5 mm adhesive tape was initially placed on the bottom of each forepaw. The mouse was set in the beaker for up to 2 min, and the time to remove each sticker was recorded. The process was then repeated three times each to create average values.

#### 2.4.5 Morris water maze

The Morris water maze is a common test of spatial cognition, learning, and memory for rodents (Maei et al., 2009). It has been extensively validated as a measure of hippocampus function by encouraging mice to use distal, visual cues to find a hidden platform (10 cm in diameter) to stand on that has been submerged by one centimeter of opaque water (nontoxic white paint water) in a large round water tank (120 cm in diameter) (Maei et al., 2009). The test was performed for 6 days, the first 5 days were trial days in which the hidden platform remained located in the same location in the maze, and one final probe day, in which the platform was removed. During the trial days, each animal performed the test four times each day, starting in each of the different quadrants, in order to learn the location of the hidden platform relative to the visual cues. On the probe trial day, the platform was removed, and the animal’s ability to remember its location was assessed. The mouse’s behavior and motion were tracked by a camera placed above the tank, and data were analyzed for a multidimensional variety of measures that assess the mouse’s ability to find the platform and the strategy used to do so.

#### 2.4.6 Y-maze

The Y-maze is a relatively simple test of spatial memory in mice (Kraeuter et al., 2019). Although the spontaneous alternation method of testing is often used for spatial working memory, the blocked arm procedure used here is designed to focus on spatial reference memory instead (Kraeuter et al., 2019). The Y-maze has three arms (each 35 cm × 5 cm × 20 cm) arranged at 120° angles from each other. The mouse spent 2 sessions in this Y-maze test. One of the arms was initially walled off during the first training session, and the animal was allowed to explore two unblocked arms for 5 min. After all mice had undergone the training session, the wall blocking the third arm was removed, and the mice were evaluated in a testing session. A camera tracks the mouse’s movements in each of the three arms for another 5 min, counting an entry into an arm as when the mouse’s center crossed into the arm. A mouse entered the previously unexplored “novel” arm at a higher frequency than the other 2 arms, which was considered strong spatial reference memory (Kraeuter et al., 2019).

#### 2.4.7 RotaRod test

The RotaRod test is used to assess motor deficits in mice resulting from various pathologies, including TBI (Hamm et al., 1994). In this study, motor function and motor learning ability were assessed using the RotaRod apparatus (Coulbourn Instruments, Whitehall, PA, United States) over a 5-day testing period. Groups of five mice were placed on the rod simultaneously at 0 rotations per minute (rpm), and the trial was initiated by pressing the start button. The rod accelerated from 4 rpm at a constant rate (4 rpm every 30 s) until reaching 40 rpm, with a maximum trial duration of 5 min. Each trial ended when the mouse fell or when 5 min had elapsed. Testing was performed once daily for 5 consecutive days, with three trials per day per mouse and an intertrial rest period of at least 15 min. Latency to fall was recorded with a stopwatch.

### 2.5 Data analysis

Statistical analysis was performed using GraphPad Prism (GraphPad Software, Inc., La Jolla, CA, United States). Data were tested for normality using D’Agostino & Pearson normality test and Shapiro–Wilk normality test. When the data were normally distributed, a one-way ANOVA with Tukey’s *post hoc* test was used to analyze differences among multiple groups. A two-way repeated-measures ANOVA, followed by Tukey’s, Dunnett’s, Sidak’s, or Bonferroni’s *post hoc* test as appropriate, was used to assess two-factor-related differences (e.g., time and housing condition). Data were presented as mean ± SEM.

## 3 Results

### 3.1 Sensorimotor performance

#### 3.1.1 Cylinder test results

**Figure 3:**
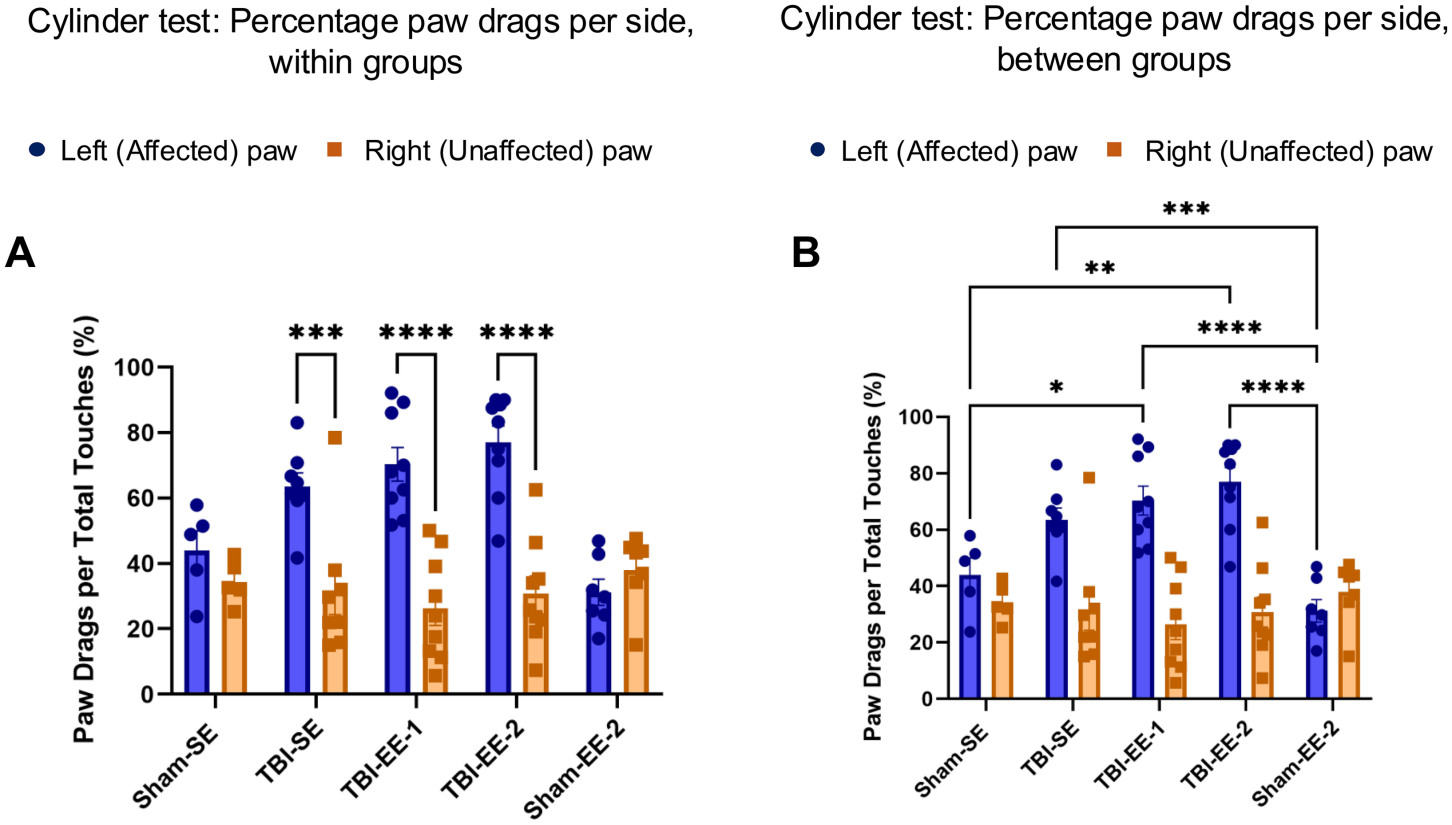
Cylinder test data. Voluntary motor function is evaluated using the cylinder test, measuring paw touches and paw drags. Performance is calculated as the percentage of paw drags per touch on both the affected and unaffected sides. **(A)** Percentage of paw drags per side within groups (two-way ANOVA with Sidak’s *post hoc* test). **(B)** Percentage of paw drags per side between groups (two-way ANOVA with Tukey’s *post hoc* test). Bars: mean ± SEM. ^*^p < 0.05, ^**^p < 0.01, ^***^p < 0.001, and ^****^p < 0.0001. Sham-SE: *n* = 5, TBI-SE: *n* = 8, TBI-EE-1: *n* = 9, TBI-EE-2: *n* = 9, Sham-EE-2: *n* = 7.

The cylinder test was the first behavioral assay used to assess somatic sensation and motor function. The percentage of paw drags relative to total wall touches made with the affected (left) paw versus the unaffected (right) paw per group was analyzed using two-way ANOVA. Significant effects were found for group (*F*_4,66_ = 4.147, *p* = 0.0047), paw (*F*_1,66_ = 52.96, *p* < 0.0001), and group × paw interaction (*F*_4,66_ = 9.085, *p* < 0.0001). *Post hoc* Sidak’s multiple-comparison tests were conducted between the affected and unaffected paws within each group (Figure 3A). Neither the Sham-SE (*p* = 0.8269) nor Sham-EE-2 (*p* = 0.9134) groups showed a significant difference between paws. In contrast, all TBI groups—TBI-SE (*p* = 0.0002), TBI-EE-1 (*p* < 0.0001), and TBI-EE-2 (*p* < 0.0001)—exhibited significant asymmetry, indicating impaired forelimb use on the affected side.

A Tukey’s *post hoc* test was performed to compare differences between groups for each paw (Figure 3B). No significant differences were observed between the Sham-SE and TBI-SE groups for either the affected/left paw (*p* = 0.1432) or the unaffected/right paw (*p* = 0.9978). Likewise, no significant differences were detected between TBI-SE and TBI-EE-1 (affected paw, *p* = 0.8753; unaffected paw, *p* = 0.9468) or between TBI-SE and TBI-EE-2 (affected paw, *p* = 0.3340; unaffected paw, *p* > 0.9999). The TBI-EE-2 group exhibited a significantly higher percentage of paw drags than the Sham-EE-2 group on the affected side (*p* < 0.0001), but not on the unaffected side (*p* = 0.8629). Similar results were observed between TBI-EE-1 and Sham-EE-2 groups (affected paw, *p* < 0.0001; unaffected paw, *p* = 0.5185).

All TBI groups, regardless of housing condition, exhibited significantly greater percentages of paw drags on the affected side, whereas sham groups did not. Both enriched-environment TBI groups also showed higher paw-drag percentages than the Sham-EE-2 group. These findings indicate that, in the cylinder test, exposure to enriched environments did not ameliorate somatosensory or motor deficits in TBI mice.

#### 3.1.2 Sticker test results

**Figure 4:**
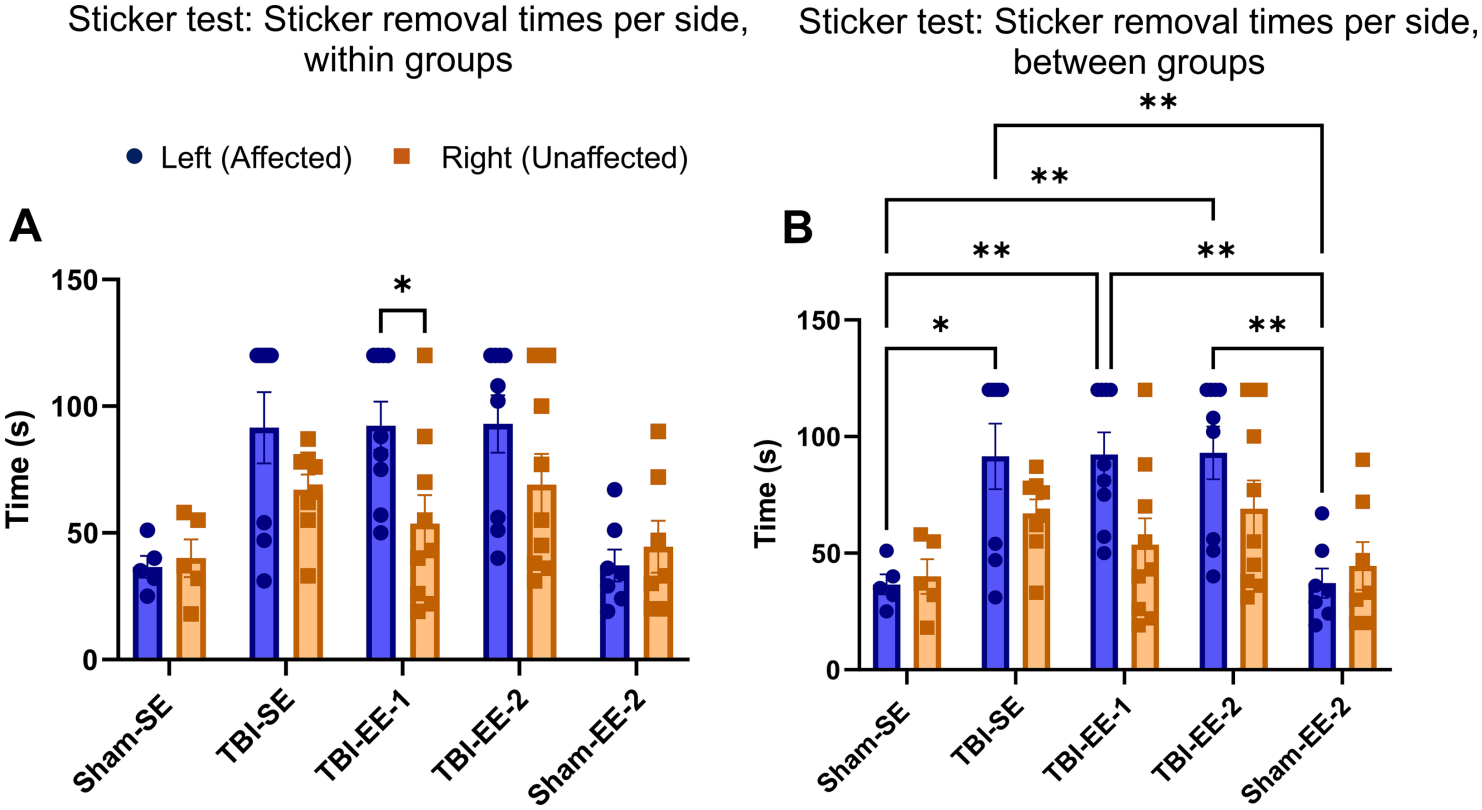
Sticker removal test data. The sticker removal test evaluates somatosensory function. Performance is measured as the mean time to recognize and remove a small sticker from the bottom of each paw. **(A)** Sticker removal times within groups. Only TBI-EE-1 mice show a significant increase in removal time for the affected paw compared to the unaffected paw (two-way ANOVA with Sidak’s *post hoc* test). **(B)** Sticker removal times between groups. Each TBI group (TBI-SE, TBI-EE-1, and TBI-EE-2) takes longer to remove stickers from the affected paws than either sham groups (Sham-SE and Sham-EE-2). No significant differences are observed among TBI groups for the affected paw, and no significant differences are observed between any groups for the unaffected paw (two-way ANOVA with Tukey’s *post hoc* test). Bars: mean ± SEM. ^*^p < 0.05 and ^**^p < 0.01. Sham-SE: *n* = 5, TBI-SE: *n* = 8, TBI-EE-1: *n* = 9, TBI-EE-2: *n* = 9, Sham-EE-2: *n* = 7.

The sticker test was also used to assess somatic sensation and motor function in mice. Similar to the cylinder test, data were analyzed using two-way ANOVA to examine differences between the affected and unaffected paws across groups. Significant main effects were observed for group (*F*_1,66_ = 4.959, *p* = 0.0294) and paw (*F*_4,66_ = 7.153, *p* < 0.0001), but not the group × paw interaction (*F*_4,66_ = 1.665, *p* = 0.1686). Sidak’s *post hoc* test revealed that the TBI-EE-1 group was the only group showing a significant difference in sticker-removal time between the affected and unaffected paws (*p* = 0.0317). Sham-SE (*p* = 0.999), TBI-SE (*p* = 0.4007), TBI-EE-2 (*p* = 0.3644), and Sham-EE-2 (*p* = 0.9935) groups all showed no significant differences between paws (Figure 4A).

Sticker-removal times were also compared between groups for each paw using Tukey’s *post hoc* test. TBI-SE mice required significantly longer times than Sham-SE mice to remove the sticker from the affected paw (*p* = 0.0129), but not from the unaffected paw (*p* = 0.4868). No significant differences were found between TBI-EE-1 and TBI-SE groups for either the affected (*p* > 0.999) or unaffected paws (*p* = 0.8795), and similar results were observed between TBI-EE-2 and TBI-SE groups (affected paw, *p* = 0.9999; unaffected paw, *p* = 0.9999). TBI-EE-2 mice took significantly longer times than Sham-EE-2 mice to remove the sticker from the affected/left paws (*p* = 0.0028), but not from the unaffected paws (*p* = 0.4585). Likewise, the TBI-EE-1 group showed longer sticker-removal times than Sham-EE-2 on the affected side (*p* = 0.0032), but not on the unaffected side (*p* = 0.9715) but not on the unaffected side (*p* = 0.9715) (Figure 4B).

Overall, the results of the sticker test were consistent with those of the cylinder test. Although fewer within-group differences were detected between the affected and unaffected paws, TBI mice across all groups performed significantly worse on the affected side compared with the corresponding sham animals. These findings indicate that enriched environments did not ameliorate somatosensory or motor deficits in severe TBI mice during the chronic phase of injury.

#### 3.1.3 RotaRod test results

**Figure 5:**
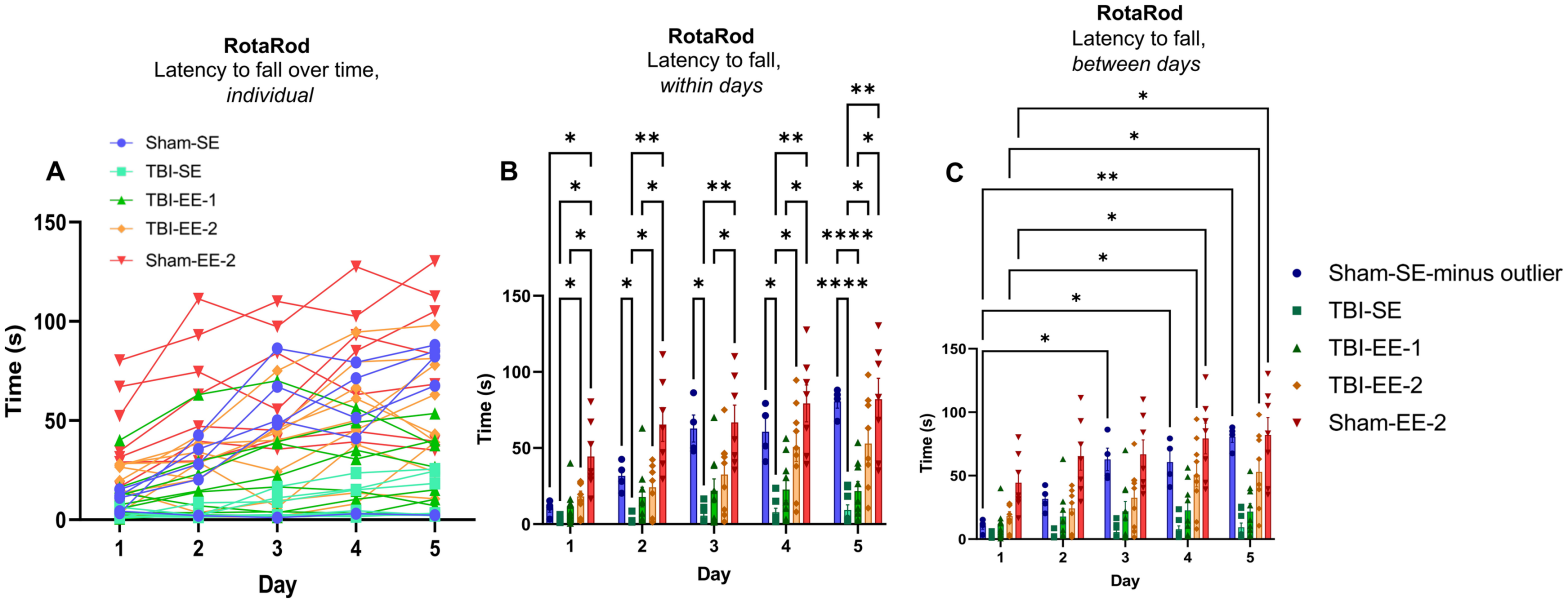
RotaRod test data. The RotaRod test evaluates motor coordination, balance, and motor learning. Mice are placed on an accelerating rotating rod, and performance is measured by latency to fall. Longer times indicate better motor function and learning. **(A)** Latency to fall over time by individual mice. Each line represents one mouse. The TBI-EE-2 and Sham-EE-2 groups exhibit a wide variation but generally show longer latencies that increase across the 5 test days. Sham-SE mice also improve across days, but start with shorter latencies on day 1, and include one non-performer who shows no improvement. TBI-SE mice display less variability than other groups and show limited improvement across the 5-day test period. **(B)** Latency to fall between groups each day, excluding the Sham-SE outlier. On all 5 days, TBI-EE-1 mice remain on the rod for significantly less time than Sham-EE-2 mice. TBI-EE-2 mice do not differ from Sham-EE-2 but perform significantly better than TBI-SE mice on all days except day 3. No significant differences are observed between TBI-EE-1 and TBI-SE mice. On days 2–5, TBI-SE mice remain on the rod for significantly shorter times than Sham-SE mice (excluding the outlier). Sham-EE-2 mice show significantly longer latencies than Sham-SE or TBI-SE mice on day 1, and longer than TBI-SE mice on all subsequent days (two-way ANOVA with Tukey’s *post hoc* test). **(C)** Latency to fall across days within groups, excluding the Sham-SE outlier. TBI-EE-2, Sham-EE-2, and Sham-SE mice (excluding the outlier) significantly increase their latency to fall by days 4 and 5 compared to day 1; Sham-SE also shows a significant increase on day 3 compared to day 1. TBI-SE and TBI-EE-1 mice show no significant improvements over the 5 days (two-way ANOVA with Dunnett’s *post hoc* test). Bars: mean ± SEM. ^*^p < 0.05, ^**^p < 0.01, and ^****^p < 0.0001. Sham-SE: *n* = 4, TBI-SE: *n* = 8, TBI-EE-1: *n* = 9, TBI-EE-2: *n* = 9, Sham-EE-2: *n* = 7.

The RotaRod test assessed not only gross motor coordination but also motor learning over time. Two-way ANOVA was performed to analyze latency to fall between groups across the five testing days. Significant effects were observed for group (*F*_4,33_ = 14.73, *p* < 0.0001), time (*F*_2.873,94.82_ = 44.58, *p* < 0.0001), and the group × time interaction (*F*_16,132_ = 5.344, *p* < 0.0001). One outlier in the Sham-SE group (Figure 5A) was excluded from the final analysis.

Tukey’s *post hoc* analysis revealed that the TBI-SE group did not differ significantly from the Sham-SE group on the first day (*p* = 0.1686), but performed significantly worse on subsequent days (day 2, *p* = 0.0333; day 3, *p* = 0.0258; day 4, *p* = 0.0273; day 5, *p* < 0.0001) compared to the Sham-SE group. No significant differences were detected between TBI-SE and TBI-EE-1 groups throughout the five testing days (day 1, *p* = 0.2474; day 2, *p* = 0.2336; day 3, *p* = 0.3188; day 4, *p* = 0.3283; day 5, *p* = 0.4339). However, TBI-SE mice remained on the rotating rod for significantly shorter durations than Sham-EE-2 mice each day (day 1, *p* = 0.0446; day 2, *p* = 0.0274; day 3, *p* = 0.0461; day 4, *p* = 0.0159; day 5, *p* = 0.0226). In contrast, TBI-EE-2 mice stayed on the rotating rod significantly longer than TBI-SE mice on all days except day 3 (day 1, *p* = 0.0104; day 2, *p* = 0.0125; day 3, *p* = 0.0564; day 4, *p* = 0.0115; day 5, *p* = 0.0110), and their performance did not differ significantly from Sham-EE-2 mice on any day (day 1, *p* = 0.1192; day 2, *p* = 0.0529; day 3, *p* = 0.1634; day 4, *p* = 0.3890; day 5, *p* = 0.4560) (Figure 5B).

Dunnett’s multiple comparisons test was used for *post hoc* analysis to evaluate motor learning within each group over the five testing days (Figure 5C). Sham-SE mice showed no significant improvement between days 1 and 2 but exhibited significant increases in latency to fall between day 1 and days 3–5 (day 1 vs. day 2, *p* = 0.1250; day 1 vs. day 3, *p* = 0.0411; day 1 vs. day 4, *p* = 0.0475; day 1 vs. day 5, *p* = 0.0048). The Sham-EE-2 group displayed a similar learning pattern, with significant improvements between day 1 and days 4 and 5 (day 1 vs. day 2, *p* = 0.0783; day 1 vs. day 3, *p* = 0.0860; day 1 vs. day 4, *p* = 0.0157; day 1 vs. day 5, *p* = 0.0191). No significant improvement was observed across days for the TBI-SE group (day 1 vs. day 2, *p* = 0.9583; day 1 vs. day 3, *p* = 0.2602; day 1 vs. day 4, *p* = 0.1945; day 1 vs. day 5, *p* = 0.1760) and the TBI-EE-1 group (day 1 vs. day 2, *p* = 0.1668; day 1 vs. day 3, *p* = 0.1475; day 1 vs. day 4, *p* = 0.1123; day 1 vs. day 5, *p* = 0.1909). However, TBI-EE-2 mice showed significant improvement over time, with longer latency to fall on days 4 and 5 compared with day 1 (day 1 vs. day 2, *p* = 0.4460; day 1 vs. day 3, *p* = 0.3224; day 1 vs. day 4, *p* = 0.0241; day 1 vs. day 5, *p* = 0.0141).

Overall, these findings indicate that the supportive enriched environment (TBI-EE-2) mitigates motor performance and motor learning deficits in severe TBI mice during the chronic phase of injury. TBI-EE-2 mice performed significantly better than TBI-SE mice on most testing days and demonstrated progressive motor learning over time. In contrast, TBI mice housed in the traditional enriched environment (TBI-EE-1) performed significantly worse than enriched sham mice (Sham-EE-2) throughout the test and were not significantly different from TBI mice in standard housing (TBI-SE), failing to demonstrate any significant motor learning over time.

### 3.2 Anxiety-related behavior

#### 3.2.1 Open field results

**Figure 6:**
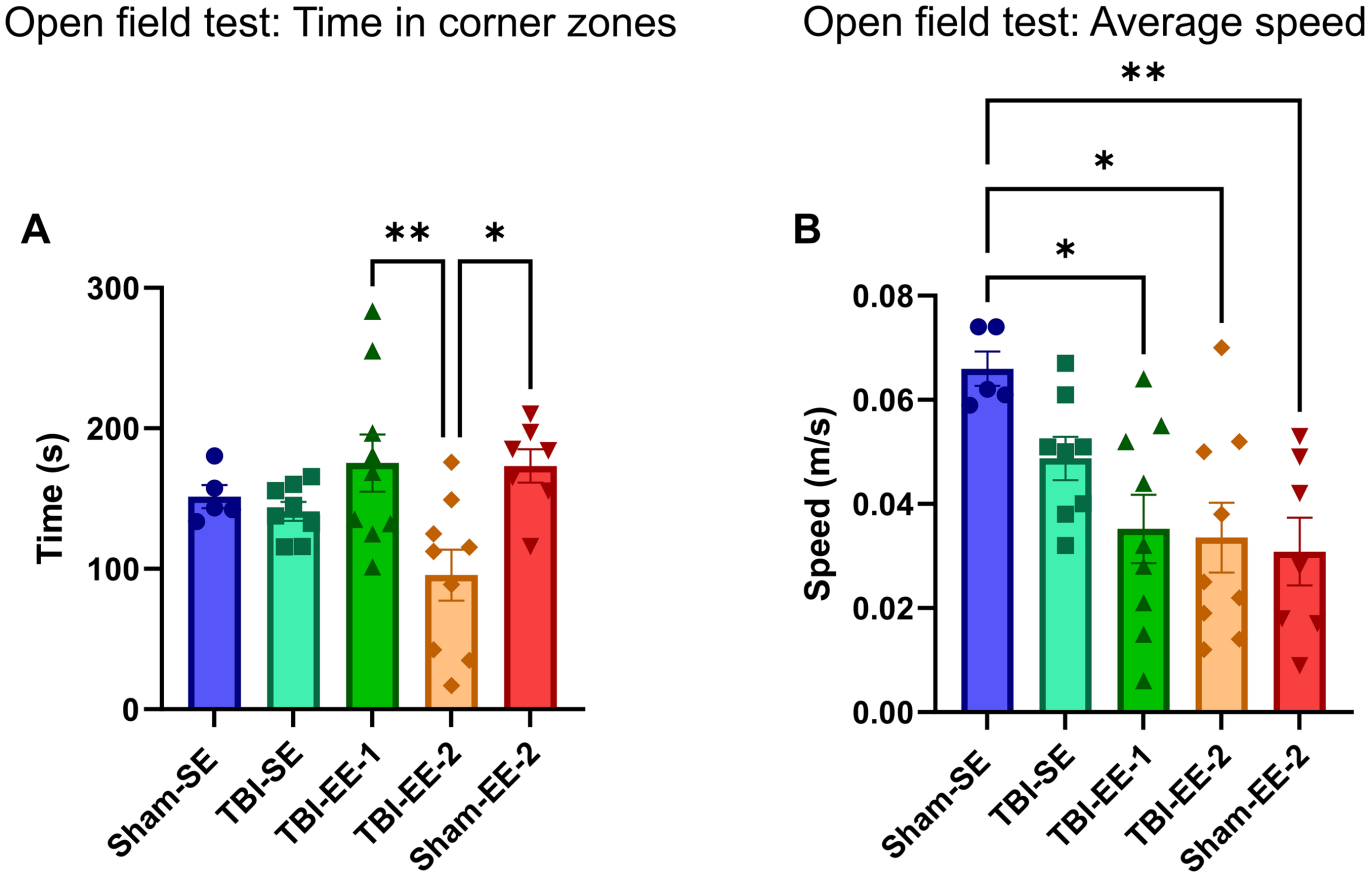
Open field test data. The open field test is used to assess anxiety-related behavior in rodents, including thigmotaxis and hyperactivity. **(A)** Mean times spent in the corner zones of the field by each group (one-way ANOVA with Tukey’s *post hoc* test). **(B)** Mean locomotor speed of mice in each group, calculated as total distance traveled per mouse over the test duration (one-way ANOVA with Tukey’s *post hoc* test). Bars: mean ± SEM. ^*^p < 0.05 and ^**^p < 0.01. Sham-SE: *n* = 5, TBI-SE: *n* = 8, TBI-EE-1: *n* = 9, TBI-EE-2: *n* = 9, Sham-EE-2: *n* = 7.

The open field test was the first behavioral assessment performed to evaluate anxiety-like behavior. Time spent in the corner zones was analyzed using a one-way ANOVA, which revealed a significant group effect (*F*_4,33_ = 4.698, *p* = 0.0041). Tukey’s *post hoc* analysis showed no significant differences between Sham-SE and TBI-SE groups (*p* = 0.9934), between TBI-SE and TBI-EE-1 groups (*p* = 0.5004), or between TBI-SE and TBI-EE-2 groups (*p* = 0.2324). However, the TBI-EE-2 group spent significantly less time in the corner zone than both the TBI-EE-1 (*p* = 0.0043) and Sham-EE-2 (*p* = 0.0107) groups (Figure 6A).

We also analyzed the speed of each group across the 10-min test. A one-way ANOVA revealed a significant effect of group (*F*_4,33_ = 4.545, *p* = 0.0049). Tukey’s *post hoc* analysis showed no significant differences in speed between Sham-SE and TBI-SE groups (*p* = 0.3894), between TBI-SE and TBI-EE-1 groups (*p* = 0.4726), between TBI-SE and TBI-EE-2 groups (*p* = 0.3568), or between TBI-EE-2 and Sham-EE-2 groups (*p* = 0.9976). Significant reductions in average speed were observed only between Sham-SE and each enriched group—TBI-EE-1 (*p* = 0.0190), TBI-EE-2 (*p* = 0.0121), and Sham-EE-2 (*p* = 0.0091) (Figure 6B).

Overall, enriched environments did not significantly alter TBI-related anxiety when comparing enriched and standard-environment TBI groups. However, the consistent decrease in locomotor speed across all enriched groups (TBI and sham) relative to Sham-SE suggests that EE may modulate anxiety-like behavior more broadly. Notably, the marked reduction in corner time in the TBI-EE-2 group compared to the other enriched groups (TBI-EE-1 and Sham-EE-2) indicates that supportive EE may provide additional anxiolytic benefits in the chronic phase of severe TBI.

#### 3.2.2 Light–dark box test results

Outcomes for the light–dark box test included time spent in the light and dark sections and the number of peeks from the dark section toward the light section. Time spent in each section was analyzed using a two-way ANOVA, which revealed a significant main effect of section (*F*_1,33_ = 81.10, *p* < 0.0001), but no significant effect of group (*F*_4,33_ = 0.000, *p* > 0.9999) or group × section interaction (*F*_4,33_ = 1.091, *p* = 0.3770).

**Figure 7:**
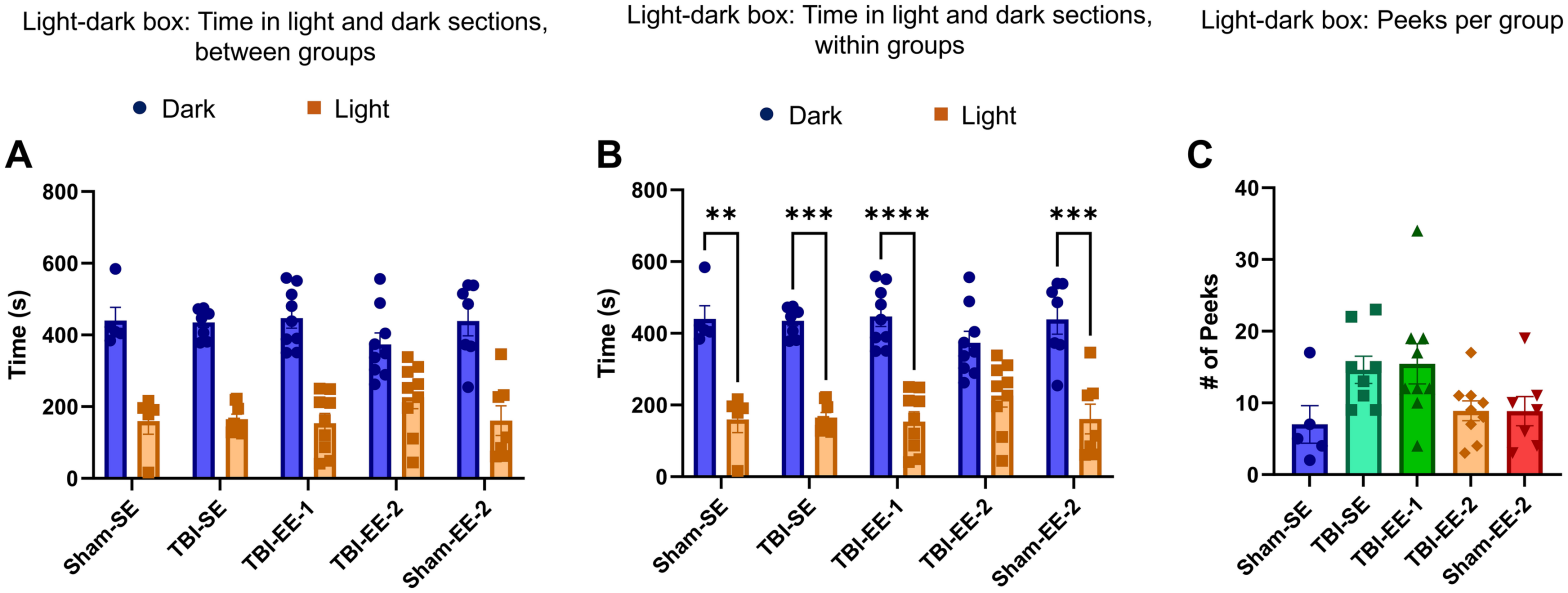
Light–dark box test data. The light–dark box test assesses anxiety-related behavior based on rodents’ avoidance of bright, open spaces. **(A)** Time spent in the light and dark sections between groups (two-way ANOVA with Tukey’s *post hoc* test). **(B)** Time spent in the light and dark sections within groups (two-way ANOVA with Bonferroni *post hoc* test). All groups, except for TBI-EE-2, spend significantly more time in the dark section than in the light section. **(C)** Number of peeks and transitions by group (one-way ANOVA with Tukey’s *post hoc* test). Bars: mean ± SEM. ^**^p < 0.01, ^***^p < 0.001, and ^****^p < 0.0001. Sham-SE: *n* = 5, TBI-SE: *n* = 8, TBI-EE-1: *n* = 9, TBI-EE-2: *n* = 9, Sham-EE-2: *n* = 7.

Tukey’s *post hoc* test showed no significant differences among any groups for either dark-section time (Sham-SE vs. TBI-SE, *p* > 0.9999; TBI-SE vs. TBI-EE-1, *p* = 0.9986; TBI-SE vs. TBI-EE-2, *p* = 0.5724; TBI-EE-1 vs. Sham-EE-2, *p* = 0.9998; TBI-EE-2 vs. Sham-EE-2, *p* = 0.5468) or light-section time (Sham-SE vs. TBI-SE, *p* > 0.9999; TBI-SE vs. TBI-EE-1, *p* = 0.9986; TBI-SE vs. TBI-EE-2, *p* = 0.5724; TBI-EE-1 vs. Sham-EE-2, *p* = 0.9998; TBI-EE-2 vs. Sham-EE-2, *p* = 0.5468) (Figure 7A).

Bonferroni multiple comparisons were used to evaluate within-group differences between time spent in the dark and light sections. Sham-SE (*p* = 0.0039), Sham-EE-2 (*p* = 0.0006), TBI-SE (*p* = 0.0004), and TBI-EE-1 (*p* < 0.0001) each spent significantly more time in the dark than in the light. In contrast, the TBI-EE-2 group did not show a significant preference for either section (*p* = 0.0691) (Figure 7B).

The number of peeks per group was analyzed using a one-way ANOVA (*F*_4,33_ = 2.894, *p* = 0.0370). Tukey’s *post hoc* test revealed no significant differences between any groups (Sham-SE vs. TBI-SE, *p* = 0.2062; TBI-SE vs. TBI-EE-1, *p* = 0.9986; TBI-SE vs. TBI-EE-2, *p* = 0.3179; TBI-EE-1 vs. Sham-EE-2, *p* = 0.2253; and TBI-EE-2 vs. Sham-EE-2, *p* > 0.9999) (Figure 7C).

Overall, these results suggest that the supportive enriched environment may reduce anxiety-like behavior in the TBI-EE-2 group, as it was the only group that did not significantly prefer the dark section over the light. However, this effect was not observed in sham animals, and the TBI-EE-2 group did not differ significantly from other groups in the absolute amount of time spent in either section.

### 3.3 Spatial learning and memory performance

#### 3.3.1 Morris water maze results

Two primary outcome measures were analyzed in the water maze: (1) travel distance and latency to reach the hidden platform during the 5-day acquisition trials and travel distance to reach the platform zone during the probe test, and (2) percentage of time spent in the edge zone of the tank during both phases.

Travel distance across the 5 days was analyzed using a two-way ANOVA, which revealed significant effects of group (*F*_4,33_ = 6.517, *p* = 0.0006), time (*F*_3.540,116.8_ = 41.26, *p* < 0.0001), and a significant group × time interaction (*F*_16,132_ = 3.041, *p* = 0.0002).

**Figure 8:**
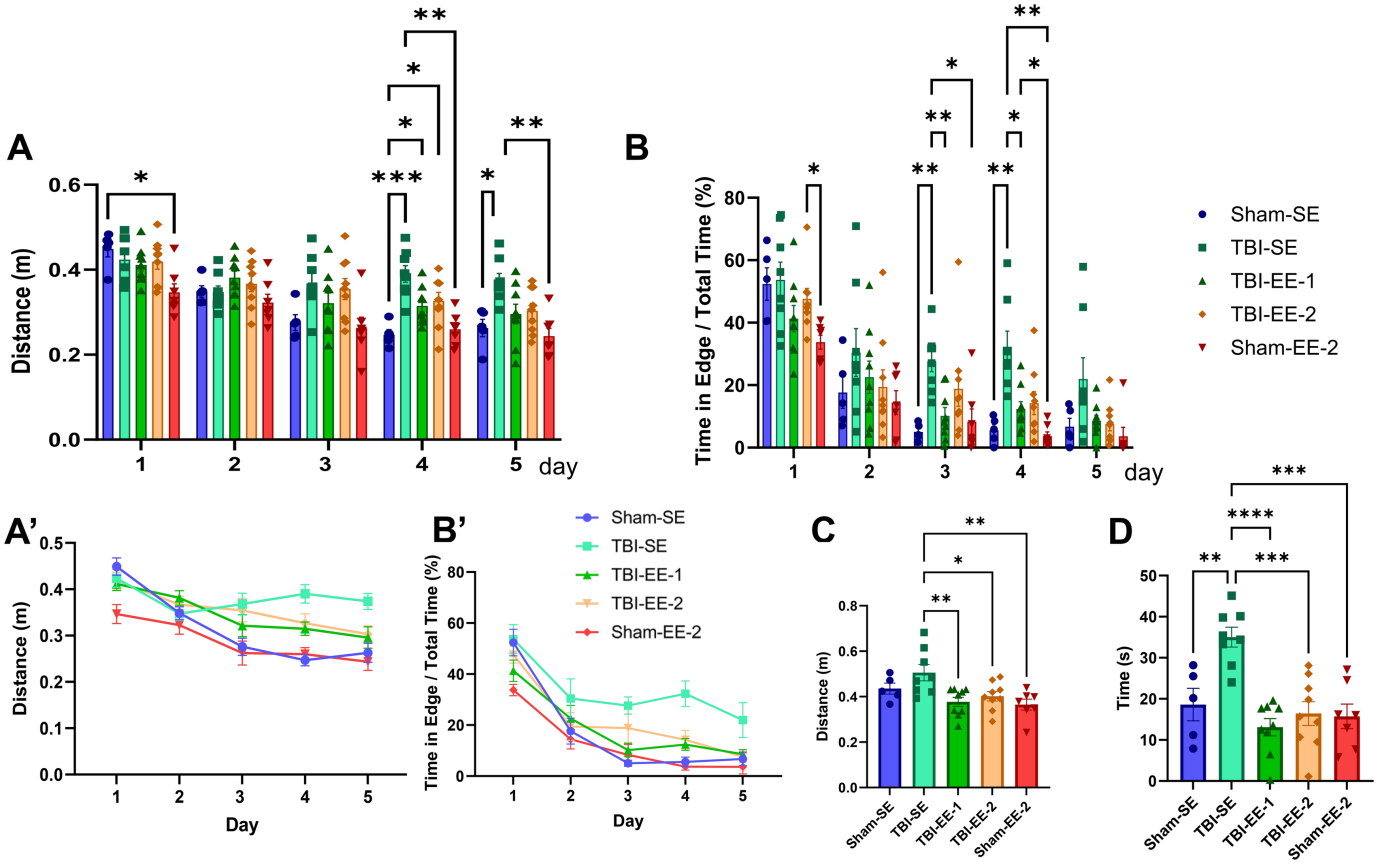
Morris water maze data. The Morris water maze test evaluates spatial learning and memory. Mice are trained to locate a hidden platform submerged in opaque water using visual cues. Performance is measured during training trials by mean distance to the platform (shorter distances reflect better spatial learning/memory) and by time spent along the water tank edge (edge zone), which reflects search strategy and learning efficiency. In addition, a probe trial without the platform is conducted to assess memory retention, measured by distance traveled to the former platform zone and by time spent in the edge zone. **(A)** Mean distance to the hidden platform across 5 trial days, between groups. On days 4 and 5, TBI-SE mice travel significantly longer distances than sham groups (Sham-SE, Sham-EE-2). On day 4, both enriched TBI groups (TBI-EE-1, TBI-EE-2) also travel longer distances than Sham-SE but not Sham-EE-2 mice (two-way ANOVA with Tukey’s *post hoc* test). **(A′)** Mean distance to the hidden platform over trial days. Distances decrease for all groups except TBI-SE. **(B)** Percentage time in the edge zone across 5 trial days, between groups. On day 1, TBI-EE-2 mice spend more time in the edge zone than Sham-EE-2 mice; this difference is not seen on later days. On days 3 and 4, TBI-SE mice spend significantly more time along the edge than Sham-SE and Sham-EE-2 mice, while TBI-EE-1 mice spend less time there than TBI-SE mice. On day 4, Sham-EE-2 mice also spend less time in the edge zone than TBI-EE-1 mice (two-way ANOVA with Tukey’s *post hoc* test). **(B′)** Percentage time in the edge zone over trial days. Edge-zone time decreases for all groups except TBI-SE. **(C)** Probe trial: distance traveled before reaching the platform zone. Enriched groups (TBI-EE-1, TBI-EE-2, Sham-EE-2) travel significantly shorter distances than TBI-SE mice. No differences are observed among the enriched groups or between Sham-SE and TBI-SE mice (one-way ANOVA with Tukey’s *post hoc* test). **(D)** Probe trial: percentage time in the edge zone. TBI-SE mice spend significantly more time in the edge zone than Sham-SE, TBI-EE-1, TBI-EE-2, and Sham-EE-2 mice. No significant differences are observed among enriched groups (TBI-EE-1, TBI-EE-2, Sham-EE-2) (one-way ANOVA with Tukey’s *post hoc* test). Bars: mean ± SEM. ^*^p < 0.05, ^**^p < 0.01, ^***^p < 0.001, and ^****^p < 0.0001. Sham-SE: *n* = 5, TBI-SE: *n* = 8, TBI-EE-1: *n* = 9, TBI-EE-2: *n* = 9, Sham-EE-2: *n* = 7.

Tukey’s *post hoc* analysis showed that Sham-SE and TBI-SE mice did not differ significantly on days 1–3 (day 1, *p* = 0.8750; day 2, *p* > 0.9999; day 3, *p* = 0.0644) but were significantly different on days 4 and 5 (day 4, *p* = 0.0006; day 5, *p* = 0.0162). TBI-EE-1 mice did not differ from TBI-SE mice on any day (day 1, *p* = 0.9840; day 2, *p* = 0.5243; day 3, *p* = 0.6418; day 4, *p* = 0.0561; day 5, *p* = 0.1049). Similarly, no significant differences were observed between TBI-SE and TBI-EE-2 across all days (day 1, *p* = 0.9998; day 2, *p* = 0.9181; day 3, *p* = 0.9943; day 4, *p* = 0.2148; day 5, *p* = 0.0790). TBI-EE-1 vs. Sham-EE-2 comparisons were also non-significant for all days (day 1, *p* = 0.1305; day 2, *p* = 0.1971; day 3, *p* = 0.4768; day 4, *p* = 0.1006; day 5, *p* = 0.4370), and the same pattern was observed for TBI-EE-2 vs. Sham-EE-2 (day 1, *p* = 0.1131; day 2, *p* = 0.5019; day 3, *p* = 0.1317; day 4, *p* = 0.1028; day 5, *p* = 0.2012) (Figures 8A,A′).

Mean travel distance was also analyzed using Dunnett’s *post hoc* test comparing each day to day 1. Sham-SE mice showed significant reductions in travel distance on all subsequent days (days 1 vs. 2, *p* = 0.0077; days 1 vs. 3, *p* = 0.0018; days 1 vs. 4, *p* = 0.0011; days 1 vs. 5, *p* = 0.0001). TBI-SE mice showed significant decreases only from day 1 to day 2 (*p* = 0.0227) and day 5 (*p* = 0.0313), with no significant improvement on day 3 (*p* = 0.2867) or day 4 (*p* = 0.4721). TBI-EE-1 mice showed significant improvement on all days except day 2 (days 1 vs. 2, *p* = 0.4129; days 1 vs. 3, *p* = 0.0422; days 1 vs. 4, *p* = 0.0003; days 1 vs. 5, *p* = 0.0062). TBI-EE-2 mice improved significantly on all days except day 3 (days 1 vs. 2, *p* = 0.0048; days 1 vs. 3, *p* = 0.0620; days 1 vs. 4, *p* = 0.0096; days 1 vs. 5, *p* = 0.0043). Sham-EE-2 mice improved significantly between days 1 and 3 (*p* = 0.0117) and days 1 and 5 (*p* = 0.0061) but not on days 2 (*p* = 0.2363) or 4 (*p* = 0.1033) (Supplementary Figure S1A).

Latency to reach the hidden platform showed patterns similar to travel distance. A two-way ANOVA followed by Tukey’s *post-hoc* test revealed significant effects of group (*F*_4,33_ = 5.051, *p* = 0.0027), time (*F*_3.365,111.0_ = 31.26, *p* < 0.0001), and group × time interaction (*F*_16,132_ = 2.298, *p* = 0.0053). Multiple comparison results are shown in Supplementary Figures S1B–D.

Time spent in the edge zone, a behavior reflecting thigmotaxis and escape attempts, was also examined. Two-way ANOVA detected significant group (*F*_4,33_ = 6.111, *p* = 0.0009) and time effects (*F*_3.180,104.9_ = 85.53, *p* < 0.0001), but the group × time interaction was not significant (*F*_16,132_ = 1.392, *p* = 0.1552).

Tukey’s *post hoc* test showed that TBI-SE mice spent significantly more time in the edge zone than Sham-SE mice on days 3 (*p* = 0.0012) and 4 (*p* = 0.0053), but not on days 1 (*p* = 0.9997), 2 (*p* = 0.6446), or 5 (*p* = 0.3082). TBI-SE mice also spent more time in the edge zone than TBI-EE-1 mice on days 3 (*p* = 0.0089) and 4 (*p* = 0.0305), but not on days 1 (*p* = 0.4374), 2 (*p* = 0.9077), or 5 (*p* = 0.3927). No significant differences were found between TBI-SE and TBI-EE-2 on any day (day 1, *p* = 0.8818; day 2, *p* = 0.7678; day 3, *p* = 0.6627; day 4, *p* = 0.0737; day 5, *p* = 0.3414). TBI-EE-1 differed from Sham-EE-2 only on day 4 (*p* = 0.0417), but not on any of the other days (day 1, *p* = 0.5280; day 2, *p* = 0.7194; day 3, *p* = 0.9944; day 5, *p* = 0.6003). TBI-EE-2 mice spent significantly more time in the edge zone than Sham-EE-2 only on day 1 (*p* = 0.0271), but not on subsequent days (day 2, *p* = 0.9395; day 3, *p* = 0.5632; day 4, *p* = 0.1203; day 5, *p* = 0.8064) (Figures 8B,B′).

Dunnett’s *post hoc* analysis within each group showed significant reductions in edge-zone time over days for Sham-SE group (days 1 vs. 2, *p* = 0.0078; days 1 vs. 3, *p* = 0.012; days 1 vs. 4, *p* = 0.0015; days 1 vs. 5, *p* = 0.0008), TBI-SE group (days 1 vs. 2, *p* = 0.0086; days 1 vs. 3, *p* = 0.0083; days 1 vs. 4, *p* = 0.0096; days 1 vs. 5, *p* = 0.0081), TBI-EE-2 group (days 1 vs. 2, *p* = 0.0001; days 1 vs. 3, *p* < 0.0001; days 1 vs. 4, *p* < 0.0001; days 1 vs. 5, *p* < 0.0001), and Sham-EE-2 group (days 1 vs. 2, *p* = 0.0070; days 1 vs. 3, *p* = 0.0012; days 1 vs. 4, *p* < 0.0001; days 1 vs. 5, *p* = 0.0002). TBI-EE-1 group showed no improvement from day 1 to day 2 (*p* = 0.1167) but showed significant improvement on days 3–5 (days 1 vs. 3, *p* = 0.0020; days 1 vs. 4, *p* = 0.0004; days 1 vs. 5, *p* = 0.0003) (Supplementary Figure S1E).

One-way ANOVA revealed that travel distance to reach the platform zone during the probe test (day 6) differed significantly across groups (*F*_4,33_ = 4.891, *p* = 0.0033). Tukey’s *post hoc* test showed no significant differences between Sham-SE and TBI-SE groups (*p* = 0.4339), Sham-EE-2 and TBI-EE-1 groups (*p* = 0.9972), or Sham-EE-2 and TBI-EE-2 groups (*p* = 0.8466). However, TBI-SE showed significantly longer travel distances than both TBI-EE-1 (*p* = 0.0065) and TBI-EE-2 groups (*p* = 0.0050) (Figure 8C).

One-way ANOVA showed that time spent in the edge zone during the probe test also differed significantly across groups (*F*_4,33_ = 10.62, *p* < 0.0001). Tukey’s *post hoc* test revealed that TBI-SE mice spent significantly more time in the edge zone than all other groups (TBI-SE vs. Sham-SE, *p* = 0.0055; TBI-SE vs. TBI-EE-1, *p* < 0.0001; TBI-SE vs. TBI-EE-2, *p* = 0.0002; TBI-SE vs. Sham-EE-2, *p* = 0.0002). No significant differences were observed between TBI-EE-1 and Sham-EE-2 groups (*p* = 0.9576) or between TBI-EE-2 and Sham-EE-2 groups (*p* = 0.9998) (Figure 8D).

Overall, the probe test provides the clearest evidence of treatment effects. Both TBI-EE-1 and TBI-EE-2 mice exhibited significantly shorter travel distances and reduced thigmotaxis compared to TBI-SE mice, indicating that both traditional and supportive enriched environments mitigate severe TBI-induced long-term impairments in spatial learning and memory.

#### 3.3.2 Y-maze results

**Figure 9:**
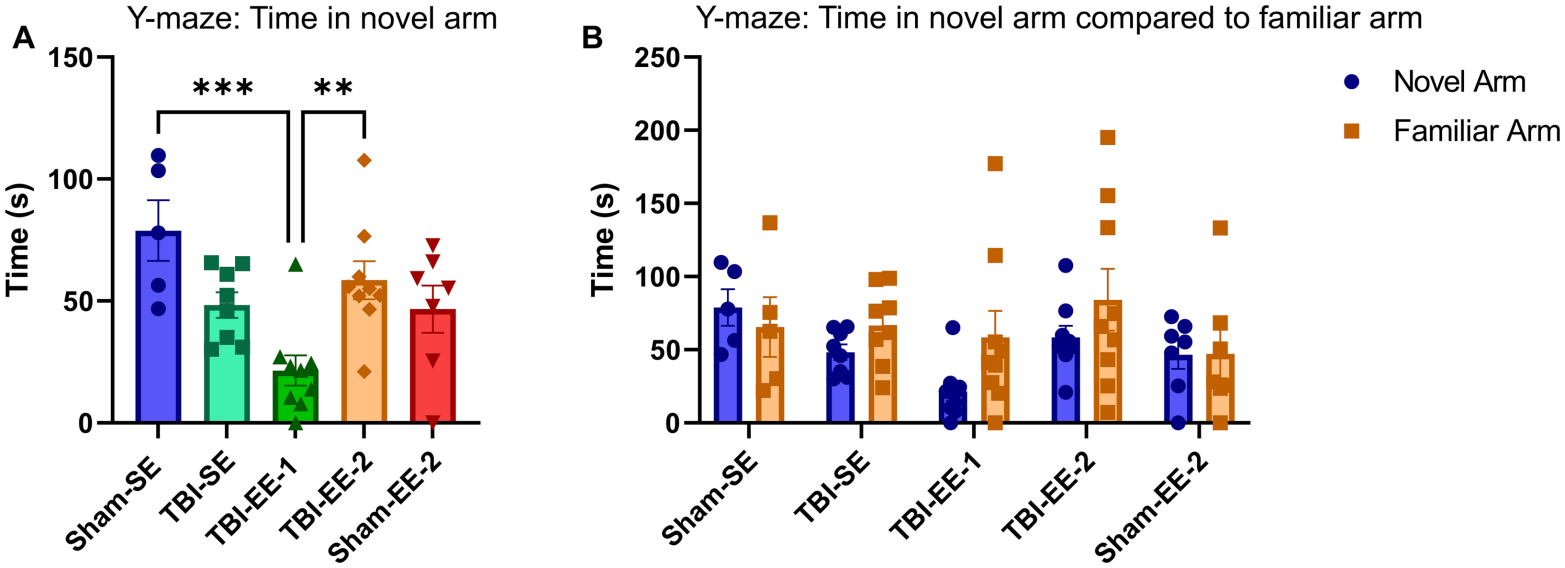
Y-maze data. The Y-maze test evaluates spatial reference/recognition memory. Mice are first familiarized with the maze while one arm is blocked, then tested for exploration of the novel arm once it is opened. Performance is measured by time spent in the novel arm compared to the familiar arms. **(A)** Time in the novel arm between groups. TBI-EE-1 mice spend significantly less time in the novel arm than TBI-EE-2 and Sham-SE mice. No other significant differences are observed (one-way ANOVA with Tukey’s *post hoc* test). **(B)** Time spent in the novel versus familiar arms for each group (two-way ANOVA with Sidak’s *post hoc* test). Bars: mean ± SEM. ^**^p < 0.01 and ^***^p < 0.001. Sham-SE: *n* = 5, Sham-EE-2: *n* = 7, TBI-SE: *n* = 8, TBI-EE-1: *n* = 9, TBI-EE-2: *n* = 9.

The spatial working memory was assessed using the Y-maze. One-way ANOVA revealed significant differences among the groups (*F*_4,33_ = 6.335, *p* = 0.0007). Tukey’s *post hoc* analysis showed that Sham-SE and TBI-SE mice did not differ in time spent in the novel arm (*p* = 0.1270). TBI-SE mice also did not differ significantly from TBI-EE-1 mice (*p* = 0.1089) or TBI-EE-2 mice (*p* = 0.8701). Neither enriched TBI group differed significantly from Sham-EE-2 (TBI-EE-1 vs. Sham-EE-2, *p* = 0.1757; TBI-EE-2 vs. Sham-EE-2, *p* = 0.8152). However, TBI-EE-1 mice spent significantly less time in the novel arm than TBI-EE-2 mice (*p* = 0.0086) (Figure 9A).

Time spent in the novel versus familiar arms was next analyzed using a two-way ANOVA. No significant effects were observed for arm (*F*_1,66_ = 2.288, *p* = 0.1352), group (*F*_4,66_ = 2.170, *p* = 0.0819), or arm × group interaction (*F*_4,66_ = 0.9207, *p* = 0.4573). Sidak’s *post hoc* comparisons showed no significant group differences for either the novel arm (Sham-SE vs. TBI-SE, *p* = 0.6288; TBI-SE vs. TBI-EE-1, *p* = 0.6013; TBI-SE vs. TBI-EE-2, *p* = 0.9816; TBI-EE-1 vs. Sham-EE-2, *p* = 0.6887; TBI-EE-2 vs. Sham-EE-2, *p* = 0.9718) or familiar arm (Sham-SE vs. TBI-SE, *p* > 0.9999; TBI-SE vs. TBI-EE-1, *p* = 0.9914; TBI-SE vs. TBI-EE-2, *p* = 0.8800; TBI-EE-1 vs. Sham-EE-2, *p* = 0.9771; TBI-EE-2 vs. Sham-EE-2, *p* = 0.3157). Tukey’s *post hoc* comparisons also revealed no significant arm preference within any group (Sham-SE, *p* = 0.9872; TBI-SE, *p* = 0.8750; TBI-EE-1, *p* = 0.2047; TBI-EE-2, *p* = 0.5816; Sham-EE-2, *p* > 0.9999) (Figure 9B).

Time spent in each arm and the center zone was then analyzed using a two-way ANOVA, which showed significant effects of zone (*F*_3,132_ = 13.16, *p* < 0.0001) and a significant zone × group interaction (*F*_12,132_ = 6.575, *p* < 0.0001), but no significant group effect (*F*_4,132_ = 0.5771, *p* = 0.6797). Tukey’s *post hoc* analysis indicated that neither Sham-SE nor TBI-EE-2 mice showed a significant preference for any area of the maze. TBI-SE mice spent significantly more time in the center zone than in the novel arm (*p* = 0.0099). Both TBI-EE-1 mice (starting arm vs. familiar arm, *p* < 0.0001; starting arm vs. novel arm, *p* < 0.0001; starting arm vs. center zone, *p* < 0.0001) and Sham-EE-2 mice (starting arm vs. familiar arm, *p* = 0.0110; starting arm vs. novel arm, *p* = 0.0104; starting arm vs. center zone, *p* = 0.0013) spent significantly more time in the starting arm than all other areas (Supplementary Figure S2A).

Group comparisons using Tukey’s *post hoc* test revealed that no group differed significantly in time spent in either the familiar or novel arms. However, TBI-EE-1 mice spent significantly more time in the starting arm than all other groups (TBI-EE-1 vs. Sham-SE, *p* < 0.0001; TBI-EE-1 vs. TBI-SE, *p* < 0.0001; TBI-EE-1 vs. TBI-EE-2, *p* < 0.0001; TBI-EE-1 vs. Sham-EE-2, *p* = 0.0062). TBI-SE mice spent significantly more time in the center zone than TBI-EE-1 (*p* = 0.0003) and Sham-EE-2 mice (*p* = 0.0020) (Supplementary Figure S2B).

Overall, these results indicate that enriched environments did not significantly improve spatial working memory after severe TBI. However, the TBI-EE-2 mice spent significantly more time in the novel arm than the TBI-EE-1 mice, suggesting a modest benefit of the supportive EE condition.

Although these results indicate that the enriched environments do not change spatial working memory in severe BI mice, there was a notable, significant difference between TBI-EE-1 and TBI-EE-2 groups, with the TBI-EE-1 mice spending significantly less time in the novel arm than the TBI-EE-2 mice. Additionally, both TBI-EE-1 and Sham-EE-2 groups spent disproportionately more time in the starting arm, particularly TBI-EE-1 mice, indicating reduced exploratory behavior. This pattern, consistent with the open field findings, suggests that enriched mice were less inclined to explore the Y-maze. In contrast, the TBI-EE-2 group’s increased time in the novel arm supports the possibility that supportive EE may confer advantages for spatial working memory after severe TBI.

## 4 Discussion

In this study, we implemented a 7-month SE period before introducing EE to model chronic severe TBI, a stage where long-term neurological impairments are well established and spontaneous recovery is minimal. Previous longitudinal studies have demonstrated that standard housing fails to reverse chronic neurodegeneration, white matter damage, or somatosensory, motor, and cognitive impairments after severe TBI (Pischiutta et al., 2018; Toshkezi et al., 2018; Qiu et al., 2020; Qiu et al., 2023). Our intention was not to re-examine these known structural and cellular changes, but rather to address a critical unanswered question: whether neurological function in the chronic phase of severe TBI can be improved through delayed EE or supportive EE.

The main findings of the present study demonstrate that supportive enriched environments improve motor coordination, motor learning, spatial memory, and anxiety-like behavior during the chronic phase of severe TBI.

The most notable improvement is observed from the RotaRod test, in which mice in the TBI-EE-2 experimental group remain on the rotating rod significantly longer than the TBI-SE control group, demonstrating improved motor coordination function at both the beginning and ends of the trials. TBI-EE-2 was also the only TBI group in the RotaRod test to exhibit significant motor learning across the 5-day testing. By contrast, TBI animals in the traditional enriched environment (i.e., TBI-EE-1) did not perform better in motor coordination than TBI-SE mice during the 5-day testing, instead having consistently worse motor coordination function than the Sham-EE-2 control group, and they did not show any significant evidence of motor learning over the 5 days. These findings highlight that, in this study, only the supportive environment containing sham mice alongside the severe TBI mice can enhance the recovery of motor coordination and motor learning during the chronic phase, while the traditional enriched environment containing only severe TBI mice fails to show these improvements.

This observation not only differs from prior literature showing improved motor function in rodents in traditional EE models, but also raises the question of what mechanism might connect the interaction with healthy individuals to better motor outcomes in chronic TBI mice (de Witt et al., 2011; Cheng et al., 2012). One potential link may be that the Sham-EE-2 mice demonstrate better exploration and play in their enriched cage, encouraging the TBI-EE-2 animals to practice these behaviors on their own more than TBI-EE-1. Also of note is that the RotaRod was the final test of the neurobehavioral battery, and that the TBI-EE-2 animals had the most time to benefit from supportive EE compared to the other tests. It is possible that the full effects of supportive EE may have taken longer than 6 weeks to appear, possibly explaining the lack of similar results in early tests.

Other neurofunctional tests illustrate differences in anxiety levels between TBI-EE-1 and TBI-EE-2. In the open field test, TBI-EE-2 mice spent significantly less time in the corners of the open field than either TBI-EE-1 or Sham-EE-2. Similarly, TBI-EE-2 is the only group of the 5 not to show a significant preference for the dark half of the light–dark box. Conventional analysis of both tests reveals that TBI-EE-2 mice show decreased anxiety around open, well-lit spaces in relation to the mice’s propensity to explore new surroundings (Seibenhener and Wooten, 2015; Bourin and Hascoët, 2003). However, TBI-EE-2 mice’s anxiety is not only reduced compared to the other TBI groups, but also to the Sham-EE-2 group in both cases. The TBI-EE-2 mice are thus implied to have an anxiety level below the baseline demonstrated by comparable healthy animals housed in the same environment. Given the well-studied association between TBI and increased anxious behaviors in both mice and humans, it is unexpected that TBI would be the sole independent variable to explain TBI-EE-2’s unique improved behavior (Dams-O’Connor et al., 2023; Sundstrøm et al., 2020; Schreiber et al., 2014). However, it may match the disinhibition seen in some human TBI patients (Glenn and Shih, 2020). Furthermore, TBI-EE-1 mice in the non-supportive traditional EE may have shown an opposite pattern of behavior in the Y-maze. Animals in the TBI-EE-1 group spent the least amount of time in the novel arm out of all the groups, preferring to remain in the starting arm for the majority of the test duration. These findings suggest a lack of spatial reference memory, but they may also be the consequence of increased anxiety in the context of exploring new environments. Regardless, the TBI-EE-2 animals’ exploratory behaviors raise interesting questions about the role of social integration with healthy mice in attenuating TBI-related anxiety.

Both supportive and traditional EE are beneficial for the severe TBI mice in long-term spatial memory. In the Morris water maze probe test, TBI-EE-1 and TBI-EE-2 mice performed better than TBI-SE mice in terms of average distance away from platform zone and time spent along the edge of the water tank. As the Morris water maze is one of the most common neurobehavioral tests for the evaluation of spatial learning and memory in rodents, these results are strongly supported by the current literature (Bondi et al., 2014; de Witt et al., 2011; Cheng et al., 2012; Lajud et al., 2019). Living in a larger, more enriching environment may have helped the TBI mice in EE cages better navigate the hidden platform in the water maze and use the surrounding visual cues without becoming overwhelmed.

The findings from this study have meaningful implications for clinical practice. The results discussed above suggest that the supportive enrichment, without the aid of surgical or pharmacological intervention, can enhance measurable recovery in neurological function during the chronic phase of severe TBI. TBI mice that lived in supportive EE during the chronic phase enhanced the recovery of motor coordination, motor learning, spatial reference memory, and anxiety-like behavior in comparison to the TBI mice that lived in either standard environments or the traditional EE. Translated to humans, this study can offer evidence to support physical rehabilitation as one of the most useful tools for functional recovery following TBI, even after the initial acute phase of care (Sundstrøm et al., 2020; Vanderploeg et al., 2008). This does not imply that EE or rehabilitation must be exclusive of other forms of treatment, but rather that supportive enrichment should not necessarily be minimized or dismissed for its role in enhancing recovery months after severe TBI onset. The present gap in available interventions has stimulated many promising new directions in TBI therapeutic research; future patient care should incorporate additional evidence-based practices into rehabilitation regimens (Khellaf et al., 2019; Bondi et al., 2014).

Nonetheless, current rehabilitation practices still have room for improvement (Centers for Disease Control and Prevention, 2015; Bayley et al., 2023). The different outcomes seen between TBI-EE-1 and TBI-EE-2 mice suggest that the integration of TBI individuals with healthy peers has benefits both for physical and neuropsychological recovery. Patients with TBI frequently experience challenges in societal reintegration, especially regarding employment and social participation, despite evidence that community integration enhances resilience (Dams-O’Connor et al., 2023; Semanision et al., 2024). Thus, rehabilitation programs for TBI may benefit from integrating social enrichment, such as structured group activities with both TBI and non-TBI patients, once patients have transitioned beyond intensive care. Additionally, the success of a supportive EE even when starting 7 months post-severe TBI suggests that the benefits of the supportive enrichment do not plateau following the acute phase. Sustained engagement in rehabilitation has the potential to yield additional symptom improvement in patients, but systemic barriers frequently hinder long-term participation. Group-based rehabilitation offers one strategy to alleviate financial and logistical challenges.

Limitations of this study include a lack of behavioral information on individual animals. EE mice engaged with enrichment items at liberty, and the animals likely varied in their activity levels, both on an individual basis and over the 10 weeks spent in the EE cages. Test outcomes, particularly anxiety measures, may also have been affected by sociobehavioral factors, given that bite wounds were observed in several TBI-EE animals, particularly those co-housed with all TBI mice (i.e., TBI-EE-1). Randomized male TBI and Sham mice were combined from multiple standard-environment cages and co-housed in EE cages in the present study, a practice that may have introduced additional stress and variability. Future work should attempt to avoid this approach. The absence of longitudinal analysis in the neurofunctional test battery represents a limitation of this study. Future research incorporating repeated testing across extended time points may better elucidate patterns of functional recovery. Ongoing work will focus on evaluating brain structural changes in the animals included in this project. These investigations may provide important insight into the effects of supportive EE on neuroplasticity during the chronic phase of severe TBI. Finally, supportive EE remains insufficiently studied in the literature and deserves further investigation. The findings presented here suggest that supportive EE constitutes a straightforward yet promising intervention to lessen the chronic consequences of severe TBI; however, more research is necessary to substantiate and extend these observations.

## 5 Conclusion

This study demonstrates that enriched environments promote spatial memory recovery in mice with chronic severe TBI over 6 weeks. Supportive enriched environments, involving co-housing severe TBI mice with healthy sham mice, further enhanced motor coordination, motor learning, and reduced anxiety-related behaviors. These findings highlight supportive enrichment as a promising strategy for chronic severe TBI rehabilitation, emphasizing the added value of social integration with healthy peers. In clinical contexts, incorporating extended rehabilitation timelines and structured social interaction may enhance recovery trajectories in patients with severe TBI.

## Data Availability

The original contributions presented in the study are included in the article/Supplementary material, further inquiries can be directed to the corresponding author.

## Supplementary material

SUPPLEMENTARY FIGURE S1 Morris water maze performance. **(A)** Travel distance to locate the hidden platform during the 5 trial days, comparing days for each group. Sham-SE, TBI-SE, and TBI-EE-2 groups display significantly decreased travel distances to the platform from day 1 to day 2. The Sham-SE, TBI-EE-1, and Sham-EE-2 groups show significantly decreased travel distance on day 3 vs. day 1. On day 4, Sham-SE, TBI-EE-1, and TBI-EE-2 mice show significantly reduced travel distances compared to day 1. On day 5, all groups exhibit significantly lower travel distances compared to day 1. **(B)** Latency to the hidden platform during the 5 trial days, comparing groups for each day. The enriched sham group (Sham-EE-2) exhibits significantly shorter latency compared to the Sham-SE, TBI-EE-1, and TBI-EE-2 groups, starting on day 1. These significant differences disappear on the following days. On day 4, TBI-SE mice show a significantly longer latency to the platform than Sham-SE and Sham-EE-2 mice. TBI-EE-2 mice display a significantly longer latency than Sham-SE mice on this day as well. By day 5, no groups demonstrate significant differences from each other. **(C)** Latency to the hidden platform during the 5 trial days, comparing days for each group. Sham-SE, TBI-SE, and TBI-EE-2 mice all show significantly decreased latency on day 2 compared to day 1. Sham-SE and TBI-EE-2 mice are significantly different from day 1 to day 3. On day 4, all groups except TBI-SE show a significantly decreased latency compared to day 1. On day 5, all groups show significant decreases from the first day. **(D)** Latency to the platform zone during the probe test. No significant differences are seen between all experimental groups. **(E)** Percentage time in the edge zone of the water tank during the 5 trial days, comparing days for each group. All five experimental groups show significantly reduced percentages of time in the edge zone on day 3 compared to day 1. This significant decrease holds for days 4 and 5. Bars: mean ± SEM. ^*^*p* < 0.05, ^**^*p* < 0.01, ^***^*p* < 0.001, and ^****^*p* < 0.0001. Two-way ANOVA with Tukey’s *post hoc* test for panels **(B–D)**. Dunnett’s *post hoc* test for panels **(A,E)**. Sham-SE: *n* = 5, Sham-EE-2: *n* = 7, TBI-SE: *n* = 8, TBI-EE-1: *n* = 9, TBI-EE-2: *n* = 9.

SUPPLEMENTARY FIGURE S2 Performance in the Y-maze test. The time spent by the mice in each arm of the Y-maze and in the triangular center zone was recorded for analysis. **(A)** Time spent in each arm and the center zone, comparing maze sections for each group. TBI-SE mice spent significantly more time in the center zone than in the novel arm. TBI-EE-1 and Sham-EE-2 mice spent significantly longer in the starting arm than in all other sections of the Y-maze. Sham-SE and TBI-EE-2 mice did not show significant differences in time spent across maze sections. **(B)** Time spent in each arm and the center zone, comparing groups within each maze section. TBI-EE-1 mice remained in the starting arm for significantly longer than Sham-SE, TBI-SE, TBI-EE-2, and Sham-EE-2 mice. TBI-SE mice spent significantly more time in the center zone than TBI-EE-1 and Sham-EE-2 mice. Bars: mean ± SEM. ^*^*p* < 0.05, ^**^*p* < 0.01, ^***^*p* < 0.001, and ^****^*p* < 0.0001. Two-way ANOVA with Tukey’s *post hoc* test. Sham-SE: *n* = 5, Sham-EE-2: *n* = 7, TBI-SE: *n* = 8, TBI-EE-1: *n* = 9, TBI-EE-2: *n* = 9.
